# [Retracted] CIP2A, an oncoprotein, is associated with cell proliferation, invasion and migration in laryngeal carcinoma cells

**DOI:** 10.3892/or.2025.8875

**Published:** 2025-02-06

**Authors:** Xu-Dong Chen, Shi-Xiong Tang, Jian-Hua Zhang, Li-Tao Zhang, Yao-Wen Wang

Oncol Rep 38: 1005–1012, 2017; DOI: 10.3892/or.2017.5759

Subsequently to the publication of the above paper, an interested reader has drawn to our attention that five pairs of data panels in Fig. 3A and B, showing the results of Transwell invasion and migration assay experiments, were overlapping, such that data which were intended to show the results from a number of differently performed experiments had apparently been derived from the same original sources.

Following an independent assessment of these data in the Editorial Office, the Editor of *Oncology Reports* has decided that this article should be retracted from the publication on account of a lack of confidence in the integrity of the presented data. The authors were asked for an explanation to account for these concerns, but the Editorial Office did not receive a reply. The Editor apologizes to the readership for any inconvenience caused by the retraction of this article.

